# Hsp90 Is Cleaved by Reactive Oxygen Species at a Highly Conserved N-Terminal Amino Acid Motif

**DOI:** 10.1371/journal.pone.0040795

**Published:** 2012-07-27

**Authors:** Raphaël Beck, Nicolas Dejeans, Christophe Glorieux, Mélanie Creton, Edouard Delaive, Marc Dieu, Martine Raes, Philippe Levêque, Bernard Gallez, Matthieu Depuydt, Jean-François Collet, Pedro Buc Calderon, Julien Verrax

**Affiliations:** 1 Louvain Drug Research Institute, Université catholique de Louvain (UCL), Brussels, Belgium; 2 Unité de Recherche en Biologie Cellulaire (URBC)-NARILIS, FUNDP-University of Namur, Namur, Belgium; 3 de Duve Institute, Université catholique de Louvain (UCL), Brussels, Belgium; 4 Departamento de Ciencias Quimicas y Farmaceuticas, Arturo Prat University, Iquique, Chile; University of Illinois at Chicago, United States of America

## Abstract

Hsp90 is an essential chaperone that is necessary for the folding, stability and activity of numerous proteins. In this study, we demonstrate that free radicals formed during oxidative stress conditions can cleave Hsp90. This cleavage occurs through a Fenton reaction which requires the presence of redox-active iron. As a result of the cleavage, we observed a disruption of the chaperoning function of Hsp90 and the degradation of its client proteins, for example, Bcr-Abl, RIP, c-Raf, NEMO and hTert. Formation of Hsp90 protein radicals on exposure to oxidative stress was confirmed by immuno-spin trapping. Using a proteomic analysis, we determined that the cleavage occurs in a conserved motif of the N-terminal nucleotide binding site, between Ile-126 and Gly-127 in Hsp90β, and between Ile-131 and Gly-132 in Hsp90α. Given the importance of Hsp90 in diverse biological functions, these findings shed new light on how oxidative stress can affect cellular homeostasis.

## Introduction

The 90 kDa heat shock protein (Hsp90) is a cellular chaperone that is essential for the growth of eukaryotic cells [1]. Hsp90 is one of the most abundant proteins in the cytoplasm, where it exists as two closely-related isoforms: The α isoform, which is highly inducible, and the β isoform, which is constitutive. Hsp90 is divided into three main structural domains: An N-terminal domain that binds ATP; a middle domain that binds client proteins; and a C-terminal dimerization domain that can also bind ATP [2]. The chaperone function of Hsp90 depends on the availability of ATP. Indeed, ATP binding to the N-terminal nucleotide-binding pocket and its subsequent hydrolysis by Hsp90 drive a conformational cycle that is essential for chaperone activity. However, considering that the affinity of the Hsp90 N-terminal nucleotide-binding pocket is greater for ADP than for ATP (Kd of 7.2 µM instead of 240 µM), only a small modification of the ADP/ATP ratio can affect Hsp90 function, changing the complex formed by Hsp90 and other chaperones and co-chaperones into a form that promotes client protein degradation by the ubiquitin-proteasome pathway [2]–[5].

Interestingly, Hsp90 is constitutively expressed at 2–10-fold higher levels in tumors compared to normal tissues [6]. Indeed, Hsp90 is used by cancer cells to protect various mutated and overexpressed oncoproteins (e.g., Bcr-Abl or mutated p53) from misfolding and degradation [4], [7]. Given its importance for the growth and survival of tumor cells, Hsp90 has become an attractive target for cancer therapy and many inhibitors have now entered clinical trials. Several of these inhibitors carry a quinone moiety and thus have redox-active properties. For instance, it has been reported that ansamycin antibiotics, such as geldanamycin, generate oxidative stress, which contribute to their cytotoxicity [8]. Since the precise role of oxidative stress in Hsp90 inhibition remains unclear [9], we sought to determine the direct influence of reactive oxygen species (ROS) generating systems on Hsp90 integrity. Indeed, it is well-known that ROS can oxidize proteins, leading to damage such as the modification of amino acid side chains, protein conversion to derivatives that are highly sensitive to proteolytic degradation, and even cleavage of the polypeptide chain [10].

In a previous report, we provided evidence that oxidative stress can provoke the cleavage of Hsp90 in two leukemia cell lines (K562 and KU812 cells) [11]. However, the precise mechanism leading to this cleavage was unknown. Here, we demonstrate that oxygen radicals, generated during the reaction between H_2_O_2_ and an intracellular source of ionic iron chelated to adenine nucleotides, attack and fragment the polypeptide backbone of Hsp90, leading to the disruption of its chaperoning function and the degradation of its client proteins.

## Results

### Oxidative Stress Leads to Hsp90 Cleavage

**Figure 1:**
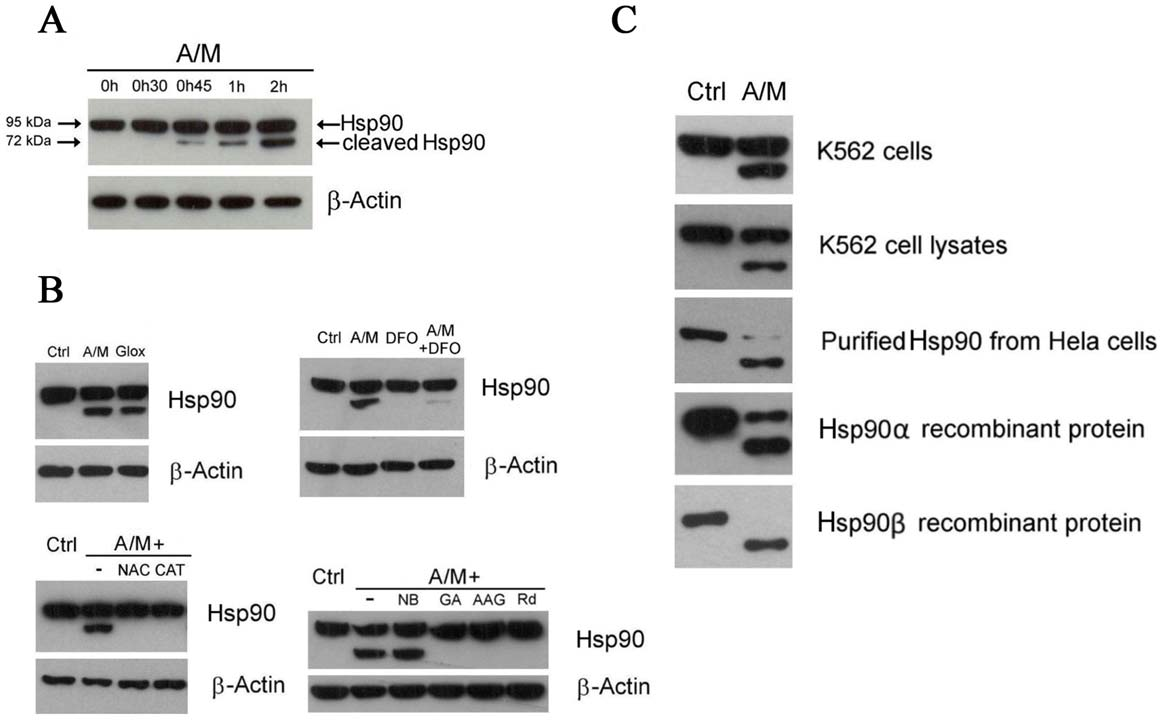
Oxidative stress leads to Hsp90 cleavage and client protein degradation. (A) Time course of Hsp90 cleavage in K562 cells upon treatment with A/M (2 mM/10 µM). Hsp90 was detected with an anti-C terminus antibody from Santa Cruz Biotechnology (Hsp90 α/β, clone F-8). (B) Hsp90 cleavage in cells incubated for 2 hours with either A/M (2 mM/10 µM) or Glox (30 mM glucose/0.25 U/ml glucose oxidase), another H_2_O_2_-generating system (top left). Suppression of Hsp90 cleavage in cells preincubated for 1 h with 3 mM N-acetylcysteine (NAC) or 100 U/ml of catalase (CAT) and then exposed to A/M for 2 h (bottom left). Iron chelation by preincubating cells with deferoxamine (DFO) for 18 h decreased the cleavage induced by 2 h treatment with A/M (top right). Preincubation of cells for 1 h with 10 µM of N-term Hsp90 inhibitors, like geldanamycin (GA), 17-AAG (AAG) or radicicol (Rd), protected Hsp90 from the cleavage induced by 2 h treatment with A/M. Novobiocin (NB) at 1 mM did not protect (bottom right). Hsp90 was detected with the same antibody as in (A). (C) Cellular Hsp90 cleavage was reproduced in K562 cell lysates, in purified Hsp90 from HeLa cells, and in Hsp90 α and β recombinant proteins. K562 cells were incubated for two hours in the absence (Ctrl) or in the presence of A/M (2 mM/10 µM). K562 cell lysates (100 µg) were incubated for 1 h in the absence (Ctrl) or in the presence of A/M supplemented with ADP (0.2 mM) and FeCl_3_ (0.5 mM). For the experiments with purified and recombinant proteins, we used 2 µg of Hsp90, incubation lasted 30 min and A/M (2 mM/10 µM) was supplemented with 0.2 mM ADP and 0.5 mM FeCl_3_. Note that Hsp90 was detected with the same antibody as in (A) with the exception of recombinant Hsp90α that was detected with an anti-penta-His antibody.

**Figure 2:**
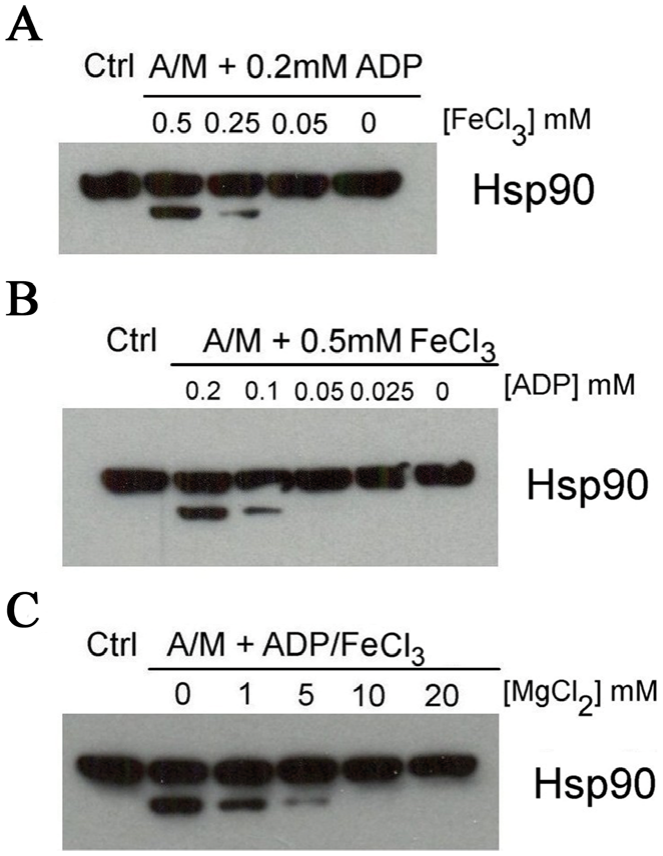
Hsp90 cleavage by A/M requires the presence of ionic iron and ADP. K562 cell lysates (100 µg) were incubated for 1 h in the absence (Ctrl) or in the presence of A/M (2 mM/10 µM) supplemented with ADP (0.2 mM) and FeCl_3_ (0.5 mM). Different concentrations of ADP (A), FeCl_3_ (B) and MgCl_2_ (C) were tested, as indicated. Hsp90 was detected with an anti-C terminus antibody from Santa Cruz Biotechnology (Hsp90 α/β, clone F-8).

In most of our experiments oxidative stress was generated during ascorbate-driven menadione redox cycling, an ROS generating system that has been extensively described by our laboratory [12]–[14]. This system is initiated by electron transfer from ascorbate (AscH^−^) to menadione (Q), as illustrated in equation [1]:
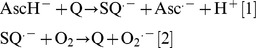



Equation [2] shows the rapid reoxidation of the semiquinone free radical (SQ^.−^) to its quinone form (Q) by molecular oxygen leading to the formation of ROS derived from superoxide anion (O_2_^.−^), such as hydrogen peroxide (H_2_O_2_) or hydroxyl radicals (HO^.^). After exposure of K562 cells to an oxidative stress generated by A/M (2 mM/10 µM), we observed partial cleavage of Hsp90 and a time-dependent formation of a C-terminal fragment of about 70 kDa, as shown in Figure 1A. Interestingly, neither a general lysosomal inhibitor (NH_4_Cl) [15], nor various protease or proteasome inhibitors were able to inhibit the cleavage induced by A/M, suggesting that the cleavage occurs through a non-enzymatic mechanism (see Figure S1). Importantly, the cleavage was not specific to A/M and similar results were obtained with other H_2_O_2_-generating systems such as glucose/glucose oxidase (Glox) (Figure 1B). Confirming the critical role of H_2_O_2_ in this process, we observed that Hsp90 cleavage induced by A/M was suppressed by addition of the antioxidant N-acetylcysteine (NAC) or catalase to the incubation medium. We also observed that deferoxamine (DFO), a potent cell-permeant iron chelator, prevented the cleavage, suggesting involvement of a Fenton-type reaction in the cleavage process (Figure 1B). Since iron can be chelated by ADP [16], and given the presence of ATP/ADP binding sites in Hsp90, we checked whether iron was carried by nucleotides in the vicinity of the peptide backbone. For this purpose, we used various Hsp90 inhibitors to block accessibility to the nucleotide binding sites and to prevent an in situ Fenton reaction. As shown in Figure 1B, geldanamycin (GA), 17-AAG, and radicicol (Rd), which all bind to the N-terminal nucleotide binding site of Hsp90 [7], suppressed the cleavage. Conversely, novobiocin (NB), which binds to the C-terminal nucleotide-binding pocket of Hsp90 [7], did not inhibit the Hsp90 cleavage induced by A/M. In K562 cell lysates, Hsp90 cleavage by A/M also required the presence of ionic iron and ADP (Figure 2A and B, respectively). In this model, we also observed that blocking the N-terminal nucleotide binding pocket with the specific inhibitors described above led to the suppression of the cleavage (not shown). Displacement of iron within the ADP-complex by adding magnesium resulted in a progressive inhibition of Hsp90 cleavage (Figure 2C). Hsp90 cleavage, with appearance of a major 70 kDa C-terminal fragment, was also observed in cellular systems, cell lysates, purified and recombinant Hsp90 proteins (Figure 1C).

### 2D-DIGE Analysis of K562 Cells after Oxidative Stress

**Figure 3:**
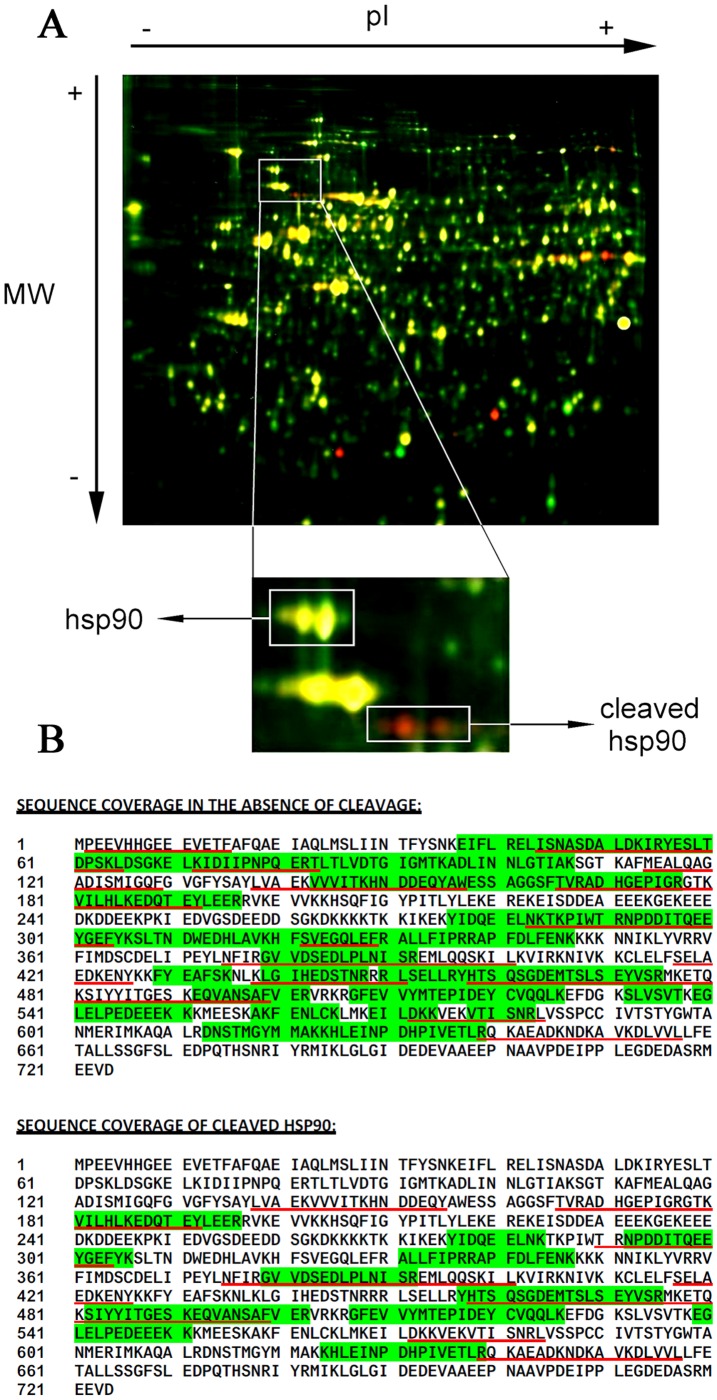
2D-DIGE analysis of protein extracts from K562 cells exposed to oxidative stress. (A) K562 cells incubated for 2 h in the absence (green) or the presence (red) of A/M (2 mM/10 µM). Spots corresponding to Hsp90 and its cleaved fragments were excised and analyzed by mass spectrometry. (B) Peptides found after mass spectrometry analysis of the excised 2D-DIGE spots of cleaved and non-cleaved Hsp90 protein by either tryptic (highlighted in green) or chymotryptic (underlined in red) peptides. Alignment was performed against the Hsp90β protein sequence.

Following two hours of A/M treatment, we detected three new spots corresponding to a molecular size of about 70 kDa (Figure 3A). Based on previous Western blots performed on 2D-gels, these spots were identified as the 70 kDa C-terminal fragment of cleaved Hsp90 (Figure S2). After preparative 2D gel electrophoresis, spots of interest were excised and submitted to in-gel proteolytic digestion with either trypsin or chymotrypsin. Several peptide fragments of either Hsp90 or its 70 kDa C-terminal fragment were generated and analyzed by Electrospray Ultra-High Resolution tandem TOF (UHR-Qq-TOF) (see Tables S1 and S2). The analysis of peptide fragments showed that Hsp90 isoforms α and β were both cleaved, but the comparative analysis took into account the amino acid sequence of Hsp90β. Sequence coverage analysis identified a region of 24 amino acids in which the cleavage must have occurred. Indeed, the peptide L138VAEKVVVITKHNDDEQY155, which was the most N-terminal peptide to be identified in cleaved Hsp90β, was immediately preceded by the peptide M114EALQAGADISMIGQF129 in uncleaved Hsp90β, suggesting that the cleavage site was located between Met-114 and Tyr-137 (Figure 3B).

### Formation of Hsp90 Protein Radicals Following Oxidative Stress

**Figure 4:**
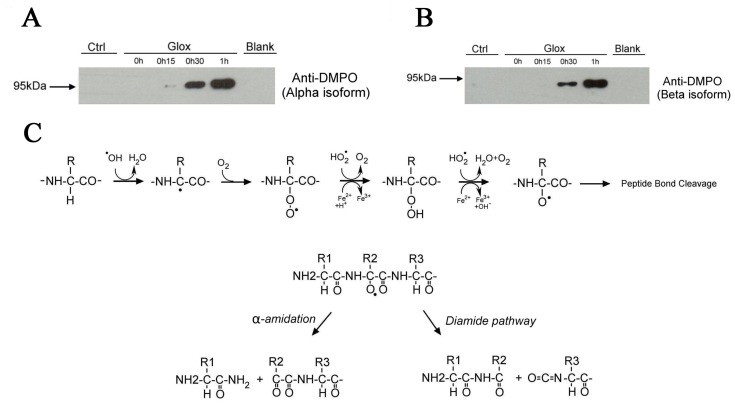
Formation of Hsp90 protein radicals following oxidative stress. (A) Immuno-spin trapping blots showing the formation of nitrone adducts in Hsp90α (200 µg) incubated with DMPO (1 mM) and exposed to oxidative stress generated by Glox (60 mM glucose/1 U/ml glucose oxidase) supplemented with ADP (0.2 mM) and FeCl_3_ (0.5 mM). Blank and Ctrl mean, respectively, that DMPO and Glox were omitted. (B) Same as (A) but with Hsp90β. (C) Proposed mechanism leading to oxidative polypeptide cleavage according to Stadman et al [18], [19].

To gain further insight into the mechanism of Hsp90 cleavage, we used the immuno-spin trapping technique for the detection of putative Hsp90 protein radicals. This technique is based on DMPO (5,5-dimethyl-1-pyrroline N-oxide), a spin trap agent, which reacts with reactive radicals to form a stable adduct that can be detected using immunoanalysis with anti-DMPO antibodies [17]. Figure 4A and 4B show the rapid formation of a nitrone adduct to both isoforms of Hsp90 (α and β) when the protein was exposed to an oxidative stress. No adduct formation was observed when Glox was omitted (Ctrl).

According to Stadtman et al [18], [19], ROS-mediated oxidation of proteins is a damaging process which can lead to cleavage of the polypeptide chain. The mechanisms of peptide bond cleavage following α-carbon oxidation are summarized in Figure 4C. Briefly, the oxidative attack of the polypeptide backbone is initiated by the HO^.−^dependent abstraction of the α-hydrogen atom from an amino acid residue to form a carbon-centered radical, which, in the presence of oxygen, is rapidly converted to an alkylperoxyl radical (ROO^.^). Further reactions with HO_2_^.^, the protonated form of superoxide anion (O_2_^.−^), or transition metals such as ferrous iron (Fe^2+^), lead to the formation of an alkoxyl radical (RO^.^) which can undergo peptide bond cleavage by either the α-amidation or diamide pathways [18], [19]. As a consequence of this mechanism, the N-terminal amino acid residue of the fragment derived from the C-terminal portion of the protein may exist either as an isocyanate (diamide pathway) or as an N-α-ketoacyl derivative (α-amidation pathway).

### Identification of the Cleavage Site

**Figure 5:**
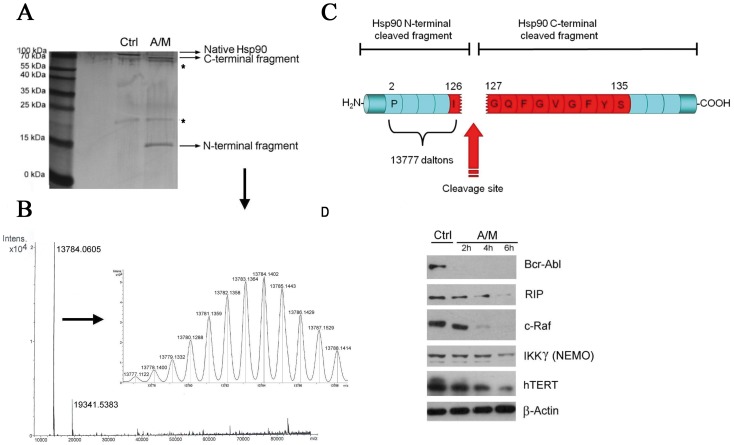
Identification of the site of cleavage within Hsp90. (A) Silver stained SDS-PAGE gel showing both C-term and N-term protein fragments of Hsp90β after 30 min incubation with A/M (2 mM/10 µM) supplemented with ADP (0.2 mM) and FeCl_3_ (0.5 mM). Asterisks show the presence of contaminants. (B) Mass spectrometry analysis of cleaved Hsp90β. Deconvoluted spectra of the small N-term fragment gave a precise monoisotopic mass of 13777.1122 daltons. (C) The picture illustrates the location of the cleavage site occurring in Hsp90β. The IGQFGVGFYS motif corresponding to a conserved amino acid sequence in several Hsp90 proteins is highlighted in red. (D) Degradation of various Hsp90 client proteins in K562 cells treated with A/M (2 mM/10 µM).

As predicted by the theoretical model presented in Figure 4C, Edman sequencing was unsuccessful because of the absence of a functional amine group at the N-terminal amino acid of the 70 kDa C-terminal fragment (not shown). As a consequence, we decided to use mass spectrometry (UHR-Qq-TOF) to identify the site of cleavage. We performed a total mass analysis of the two main fragments that were observed after the cleavage of recombinant Hsp90β by A/M (Figure 5A). The mass of the C-terminal fragment of about 70 kDa could not be measured precisely because it was too large to obtain a precise monoisotopic profile. In contrast, the precise mass of the small N-terminal fragment was successfully measured, giving a monoisotopic mass of 13777,1122 Daltons (Figure 5B). By using bioinformatic tools (GPMAW 8.21 software), we found, with a precision of 5 ppm, that such a mass corresponded to the theoretical mass of a fragment peptide starting at Pro-2 and extending to Ile-126 (Figure 5C). We can, therefore, conclude that protein cleavage was the result of a scission in the protein backbone between Ile-126 and Gly-127 in Hsp90β. It is noteworthy that hsp90α and β have their initiator methionine removed, explaining that the N-terminal fragment that we analyze begins with proline [20]. Only one other peptide could correspond to the mass measured by mass spectrometry but since it was an internal peptide (from Arg-82 to Gln-207), it was obvious that it could not be the N-terminal fragment generated by the cleavage. Interestingly, the amino acid sequence where the cleavage occurred (I126GQFGVGFYS135) has previously been identified as a conserved motif in several members of the Hsp90 family, and belongs to the N-terminal nucleotide binding site of Hsp90 [21], [22]. Finally, we noted that Hsp90 cleavage is biologically relevant, as shown in Figure 5D. Decrease in the abundance of various client proteins, including Bcr-Abl, RIP, c-Raf, IKKγ or hTert, is observed, reflecting a loss of Hsp90 chaperone function which will ultimately provoke K562 cell death (not shown).

## Discussion

This is the first study to show that ROS, formed by a Fenton-type reaction in proximity to the N-terminal nucleotide binding site of Hsp90, provoke oxidative cleavage of the polypeptide chain of Hsp90. Indeed, other studies had previously shown that chaperoning function of Hsp90 can be disrupted by protein cleavage induced by hydrogen peroxide [23], [24] or other reactive oxygen species [25] but the mechanisms underlying such a protein cleavage were unknown. In this study, the oxidative cleavage was observed in intact cells, in cell lysates and with purified and recombinant Hsp90 proteins, suggesting that proteases are unlikely to be involved. Whatever the system used, the cleavage yielded a similar C-terminal fragment of about 70 kDa. We postulate that the mechanism of oxidative cleavage relies on local generation of ROS in the Hsp90 N-terminal nucleotide-binding pocket by interaction between ionic iron (chelated with adenine nucleotides) and H_2_O_2_, generated either by ascorbate-driven menadione redox cycling (A/M) or by the glucose/glucose oxidase (Glox) system. Indeed, cleavage of H_2_O_2_ by iron (or copper) is a major source of hydroxyl radicals under physiological conditions [26].

In non-cellular systems (cell lysates, purified or recombinant protein), the protein cleavage was ADP- and iron-dependent, suggesting that ionic iron chelated with ADP was brought to the nucleotide-binding site of Hsp90. Here, it reacted with H_2_O_2_ (generated by either A/M or Glox), leading to a local generation of hydroxyl radical through a Fenton-type reaction. Interestingly, a similar system (iron-ADP plus ascorbate) has been reported in experiments performed to identify an N-terminal ATP-binding site in purified Hsp90 proteins [27], [28]. The need for iron in proximity to the protein was confirmed by competition experiments with magnesium salts (see Figure 2C). Indeed, Mg^2+^ can bind to ADP better than iron [29], [30], so its addition displaces iron from the ADP complex and, consequently, inhibits the generation of hydroxyl radical and Hsp90 cleavage. These observations are in agreement with the original description of a “site specific” Fenton mechanism in which the binding of the transition metal ions to the biological target is a prerequisite for the production of damage [31].

In cellular systems, N-acetylcysteine and catalase prevented the cleavage, indicating that oxidative stress, as a result of the generation of H_2_O_2_, is required to cleave Hsp90. In intact cells, on the contrary to non-cellular systems, any supplementation of either iron or nucleotides was worthless. However, although the intracellular source of iron was not identified, modulation of iron availability by deferoxamine, an iron chelator, also prevented Hsp90 cleavage, indicating that redox active iron is also required. The need for adenine nucleotide bound to Hsp90 was further confirmed by competition experiments with geldanamycin, radicicol and 17-AAG, all well-known Hsp90 inhibitors [7]. By binding to the N-terminal nucleotide-binding pocket of Hsp90, these compounds prevented the binding of adenine nucleotides (and therefore the putative ADP/iron complex) to the same pocket and prevented Hsp90 cleavage. We can therefore conclude from these observations that the presence of iron and nucleotides is absolutely required for the cleavage, whatever the experimental system used.

Further mass spectra analyses of the low molecular size fragment obtained by oxidative cleavage of purified Hsp90 proteins allowed us to investigate the site of cleavage within the protein. Since the precise mass of the N-cleaved fragment was successfully measured as 13777.1122 Daltons, corresponding to the molecular size of the peptide sequence from Pro-2 to Ile-126, the site of cleavage in oxidized Hsp90β must be located between Ile-126 and Gly-127, corresponding to Ile-131 and Gly-132 in Hsp90α. Interestingly, the initial amino acid sequence of the C-cleaved fragment of about 70 kDa starting at Gly-127, contains the IGQFGVGFYS motif, except for Ile-126. It should be noted that this motif (IGQFGVGFYS) has been reported as a conserved sequence in Hsp90 proteins from different species [21], [22], and several papers have shown that it belongs to the nucleotide binding site [21], [32]. Moreover, Prodromou et al [21], working with purified Hsp90 from Saccharomyces cerevisiae, reported that at least the 3 glycines and the Phe-133 are actively involved in the nucleotide binding pocket by interacting with phosphate groups and Mg^2+^. The sequence coverage of digested peptides in the full length protein and the cleaved fragment showed that the cleavage took place within a region of 24 amino acids that contains the IGQFGVGFYS motif. Interestingly, this motif did not contain side-chains of amino acid residues that can be easily oxidized by metal ion-catalyzed oxidation leading to peptide bond cleavage. Indeed, the aromatic amino acids Phe-129, Phe-133 and Tyr-134 are preferential targets for oxidation to yield hydroxyl-derivatives, while oxidation of Met-125, although not belonging to the amino acid conserved sequence but close to Ile-126, may be considered as part of a cyclic oxidation/reduction, and therefore a reversible covalent modification [19]. Since HO. reacts with almost every molecule at rate constants close to 10^9^–10^10^ M^−1^×s^−1^ [33], such a lack of discrimination implies that a precise site of cleavage at this particular region relies on both the in situ ROS formation and the specific binding of the nucleotide that carries the metal-ion catalyst. It can, therefore, be assumed that the site of cleavage in K562 cells exposed to oxidative stress should be the same as that observed with oxidized Hsp90β.

Pioneer work performed by Stadtman’s group has clearly established that oxidation of proteins by ROS can lead to the cleavage of peptide bonds either by α-amidation or diamide pathways [10], [19]. Based on the hypothetical scheme outlined in Figure 4C, the N-terminal amino acid of the fragment derived from the oxidative cleavage of the protein will exist either as an isocyanate or an N-α-ketoacyl derivative, which explains the unsuccessful assays to perform Edman sequencing for this 70 kDa C-terminal fragment. Although this assumption needs further investigation, it is likely that the C-terminal amino acid of the fragment derived from the N-terminal region of the protein (i.e. Ile-126) will exist as the amide derivative rather than a carboxylic group, since the molecular mass determined by mass spectrometry corresponds to within 0.01% to this condition. As discussed above, prior to protein cleavage, formation of a protein radical is required. The proposed mechanism of free radical attack involves abstraction of a hydrogen atom leading to the formation of a carbon-centered radical, which, in the presence of oxygen, is rapidly converted to the peroxyl radical (Figure 4C). Immuno-spin trapping analysis confirmed the formation of a protein radical when Hsp90 was incubated with an H_2_O_2_-generating system. This protein radical can be further rearranged, finally leading to protein cleavage, presumably via the α-amidation pathway.

The biological relevance of Hsp90 cleavage by oxidative stress is highlighted by the loss of its chaperone function, leading to client protein degradation. Indeed, we report here and elsewhere [11], [14] the degradation of bcr-abl, RIP, Akt, mutated bcr-abl, c-Raf, hTert and IKKγ/NEMO following the cleavage of Hsp90 by A/M. Such a loss of chaperoning function is likely explained by the removal of very important amino acid residues. Thus, Prodromou et al [21] have identified, in yeast, that eleven amino acids of the N-terminal domain participate in interactions with nucleotides, namely Leu-34, Asn-37, Asp-40, Ala-41, Asp-79, Gly-83, Met-84, Asn-92, Lys-98, Gly-118, Gly-121, Gly-123, Phe-124 and Thr-171. These amino acids correspond to Leu-43, Asn-46, Asp-49, Ala-50, Asp-88, Gly-92, Met-93, Asn-101, Lys-107, Gly-127, Gly-130, Gly-132, Phe-133 and Thr-179 in human Hsp90β. Since the cleavage of Hsp90β occurs between Ile-126 and Gly-127, it would remove 9 of the 14 residues which are critical for nucleotide binding. In addition, the cleavage would also remove Glu-42 (corresponding to Glu-33 in yeast), a catalytic glutamate residue which is required for ATPase activity [21].

In conclusion, we have observed that in situ formation of ROS by a Fenton-type reaction at the N-terminal nucleotide binding pocket of Hsp90 forms a protein radical, which, by rearrangement, causes the rupture of the peptide backbone and loss of its chaperoning function. Interestingly, we have previously demonstrated that oxidative cleavage of Hsp90 is preferentially observed in cancer cells, where it leads to cell death, likely in response to the destabilization and degradation of Hsp90 client proteins [11]. The high sensitivity of cancer cells to the oxidative cleavage of Hsp90 is probably explained by their general sensitivity towards oxidative stress, a characteristic due to frequent alterations of their antioxidant defenses and increased endogenous ROS production [34]–[36]. Oxidative cleavage of Hsp90 could, therefore, be an interesting option for killing cancer cells because this chaperone is used by cancer cells to stabilize various mutated and overexpressed oncoproteins that are critical for their growth and survival.

## Materials and Methods

### Cell Line and Chemicals

The K562 cell line (human chronic myelogenous leukaemia) was purchased from ECACC (Salisbury, United Kingdom) and maintained in RPMI medium supplemented with 10% fetal calf serum, 100 µg/ml streptomycin and 100 U/ml penicillin. Menadione sodium bisulfite, sodium ascorbate, dimethylsulfoxide, D-glucose, diethylenetriaminepentaacetic acid (DTPA), glucose oxidase, hydrogen peroxide, FeCl_3_, novobiocin, deferoxamine mesylate, catalase, protease inhibitor cocktail and radicicol were purchased from Sigma (St Louis, MO, USA). Geldanamycin (GA) and 17-allylamino-17-demethoxygeldanamycin (17-AAG) were purchased from Invivogen (San Diego, CA, USA). ADP was purchased from Roche (Basel, Switzerland). 5,5-Dimethyl-1-pyrroline N-oxide (DMPO) was purchased from Enzo Life Sciences (Farmingdale, NY, USA). Human recombinant Hsp90α was prepared as follows: Briefly, the Hsp90α insert from pcDNA3-Hsp90α plasmid (gift of Dr. Len Neckers, National Cancer Institute, Rockville, MD, USA) was cloned into the bacterial expression vector pET28a. The construct was then transferred to the E. coli BL21 and Hsp90α protein expression was induced by the addition of 1 mM isopropyl β-D-1-thiogalactopyranoside (IPTG). Recombinant protein was then purified through an Ni-NTA agarose column from Qiagen (Hilden, Germany). Human Hsp90β recombinant protein and Hsp90 purified protein from HeLa cells were purchased from Stressgen (Ann Harbor, MI, USA). All other chemicals were ACS reagent grade.

### Immunoblotting

Immunoblotting was performed as described previously [11]. Antibodies to c-Raf and c-abl were from Cell Signaling Technology (Danvers, MA, USA), antibodies to hTERT, to Hsp90 and to IKKγ (NEMO) were from Santa Cruz Biotechnology (Santa Cruz, CA, USA), antibodies to β-actin and DMPO were from Abcam (Cambridge, UK), antibodies to RIP were from Pharmingen (San Jose, CA, USA), and the penta-His antibody was from Qiagen (Hilden, Germany).

### 2D-DIGE and MS Analysis

Cells were washed with PBS and lysed in a Tris buffer (30 mM, pH 8.5) containing 7 M urea, 2 M thiourea, and 4% CHAPS. Samples were centrifuged (13,000 g, 10 min) and supernatants were kept at −80°C until use. Twenty-five micrograms of proteins from four independent samples (2 controls and 2 treated) were cross-labeled with 200 pmoles of Cy3 or Cy5 dyes. An equal volume of buffer (7 M urea, 2 M thiourea, 2% CHAPS, 0.5% IGP 4–7 buffer and 0.4% DTT) was added to each sample. First-dimension isoelectric focusing (IEF) was performed with 24-cm Immobiline dry-strips, pH 4–7 (G.E. Healthcare, Uppsala, Sweden). Strips were rehydrated according to the manufacturer’s instructions and IEF was carried out on an IPGphor IEF system (GE Healthcare), using a gradient increase in voltage (up to 8000 V) for a total of 50,000 Vh. Strips were equilibrated by two steps of 15 min, first in 1.5 M TrisHCl, 6 M urea, 30% glycerol, 2% SDS, 1% DTT, and second in the same buffer with 2.5% iodoacetamide but without DTT. SDS-PAGE was performed on 10% polyacrylamide gels. Gels were scanned with a Typhoon 9400 imager (GE Healthcare) and analyzed using DeCyder 2D (GE Healthcare). Preparative 2-D gel electrophoresis was performed using the same protocol, but with 200 µg of unlabeled proteins. Gels were stained with Krypton (Pierce, Rockford, IL, USA) and the spots of interest were excised by an automated picker (GE Healthcare).

For mass spectrometric identification, proteins in gel were digested overnight at 37°C with either trypsin or chymotrypsin (Promega, Madison, IL, USA). Peptides were analyzed on a U-3000 nanoLC (Dionex, Sunnyvale, CA, USA) equipped with an Acclaim PepMap100 nano C18 column (Dionex) and coupled to a Bruker maXis ESI Ultra-High Resolution tandem TOF (UHR-Qq-TOF) mass spectrometer (nanoLC-MSMS). Peak lists were created using Compass DataAnalysis 4.0 (Bruker, Bremen, Germany) and imported in ProteinScape 2.0 (Bruker) using a Mascot 2.2 (Matrix Science, London, UK) inhouse server. Enzyme specificity was set to trypsin or chymotrypsin and the maximum number of missed cleavages was set at 1. Carbamidomethylation and methionine oxidation were taken into account. Protein identification was performed with the NCBInr database, using a minimal individual peptide score equal or superior to the identity score. The small fragment resulting from Hsp90 cleavage was analyzed as described above, except that samples were separated on an Acclaim PepMap300 C4 column. Mass spectra were deconvoluted using Compass DataAnalysis 4.0 (Bruker) and MaxEnt for charge deconvolution. GPMAW 8.2 (Lighthouse Data, Odense, Denmark) was used to find the amino acid sequence corresponding to the monoisotopic mass measured by the maXis.

## Supporting Information

Figure S1 **Hsp90 cleavage is not suppressed by protease inhibitors.** K562 cells were preincubated for 1 h with the following compounds: MG132 (40 µM), calpeptin (50 µM), pepstatin (100 µM), antipain (50 µM), leupeptin (200 µM), and NH4Cl (5 mM), and then further exposed to A/M (2 mM/10 µM) for 2 h. Hsp90 was detected with an anti-C terminus antibody from Santa Cruz Biotechnology (Hsp90 α/β, clone F-8).
(TIF)

Figure S2 **Detection of cleaved and uncleaved Hsp90 after 2D gel electrophoresis.** Cells were incubated for 2 h in the absence (Ctrl) or in the presence of A/M (2 mM/10 µM). Cells were lysed and samples were run on 2D gels, as described in the Material and Methods section. Proteins were then transferred to PVDF membranes and Hsp90 was detected with an anti-C terminus antibody from Santa Cruz Biotechnology (Hsp90 α/β, clone F-8).
(TIF)

Table S1 **High-resolution UHR-Qq-TOF mass measurements of peptides formed by the proteolytic digestion of non-cleaved Hsp90.**
(DOC)

Table S2 **High-resolution UHR-Qq-TOF mass measurements of peptides formed by the proteolytic digestion of the 70 kDa C-terminal fragment of cleaved Hsp90.**
(DOC)

## References

[1] Borkovich KA, Farrelly FW, Finkelstein DB, Taulien J, Lindquist S (1989). hsp82 is an essential protein that is required in higher concentrations for growth of cells at higher temperatures.. Mol Cell Biol.

[2] Pearl LH, Prodromou C (2006). Structure and mechanism of the Hsp90 molecular chaperone machinery.. Annu Rev Biochem.

[3] McLaughlin SH, Ventouras LA, Lobbezoo B, Jackson SE (2004). Independent ATPase activity of Hsp90 subunits creates a flexible assembly platform.. J Mol Biol.

[4] Neckers L (2002). Hsp90 inhibitors as novel cancer chemotherapeutic agents.. Trends Mol Med.

[5] Schneider C, Sepp-Lorenzino L, Nimmesgern E, Ouerfelli O, Danishefsky S (1996). Pharmacologic shifting of a balance between protein refolding and degradation mediated by Hsp90.. Proc Natl Acad Sci U S A.

[6] Ferrarini M, Heltai S, Zocchi MR, Rugarli C (1992). Unusual expression and localization of heat-shock proteins in human tumor cells.. Int J Cancer.

[7] Trepel J, Mollapour M, Giaccone G, Neckers L (2010). Targeting the dynamic HSP90 complex in cancer.. Nat Rev Cancer.

[8] Dikalov S, Landmesser U, Harrison DG (2002). Geldanamycin leads to superoxide formation by enzymatic and non-enzymatic redox cycling. Implications for studies of Hsp90 and endothelial cell nitric-oxide synthase.. J Biol Chem.

[9] Clark CB, Rane MJ, El Mehdi D, Miller CJ, Sachleben LR (2009). Role of oxidative stress in geldanamycin-induced cytotoxicity and disruption of Hsp90 signaling complex.. Free Radic Biol Med.

[10] Stadtman ER (2006). Protein oxidation and aging.. Free Radic Res.

[11] Beck R, Verrax J, Gonze T, Zappone M, Pedrosa RC (2009). Hsp90 cleavage by an oxidative stress leads to its client proteins degradation and cancer cell death.. Biochem Pharmacol.

[12] Verrax J, Pedrosa RC, Beck R, Dejeans N, Taper H (2009). In situ modulation of oxidative stress: a novel and efficient strategy to kill cancer cells.. Curr Med Chem.

[13] Verrax J, Delvaux M, Beghein N, Taper H, Gallez B (2005). Enhancement of quinone redox cycling by ascorbate induces a caspase-3 independent cell death in human leukaemia cells. An in vitro comparative study.. Free Radic Res.

[14] Beck R, Pedrosa RC, Dejeans N, Glorieux C, Leveque P (2011). Ascorbate/menadione-induced oxidative stress kills cancer cells that express normal or mutated forms of the oncogenic protein Bcr-Abl. An in vitro and in vivo mechanistic study.. Invest New Drugs.

[15] Ohkuma S, Chudzik J, Poole B (1986). The effects of basic substances and acidic ionophores on the digestion of exogenous and endogenous proteins in mouse peritoneal macrophages.. J Cell Biol.

[16] Esterbauer H, Cheeseman KH, Dianzani MU, Poli G, Slater TF (1982). Separation and characterization of the aldehydic products of lipid peroxidation stimulated by ADP-Fe2+ in rat liver microsomes.. Biochem J.

[17] Gomez-Mejiba SE, Zhai Z, Akram H, Deterding LJ, Hensley K (2009). Immuno-spin trapping of protein and DNA radicals: “tagging” free radicals to locate and understand the redox process.. Free Radic Biol Med.

[18] Berlett BS, Stadtman ER (1997). Protein oxidation in aging, disease, and oxidative stress.. J Biol Chem.

[19] Stadtman ER, Levine RL (2003). Free radical-mediated oxidation of free amino acids and amino acid residues in proteins.. Amino Acids.

[20] Lees-Miller SP, Anderson CW (1989). Two human 90-kDa heat shock proteins are phosphorylated in vivo at conserved serines that are phosphorylated in vitro by casein kinase II.. J Biol Chem.

[21] Prodromou C, Roe SM, O’Brien R, Ladbury JE, Piper PW (1997). Identification and structural characterization of the ATP/ADP-binding site in the Hsp90 molecular chaperone.. Cell.

[22] Stebbins CE, Russo AA, Schneider C, Rosen N, Hartl FU (1997). Crystal structure of an Hsp90-geldanamycin complex: targeting of a protein chaperone by an antitumor agent.. Cell.

[23] Pantano C, Shrivastava P, McElhinney B, Janssen-Heininger Y (2003). Hydrogen peroxide signaling through tumor necrosis factor receptor 1 leads to selective activation of c-Jun N-terminal kinase.. J Biol Chem.

[24] Panopoulos A, Harraz M, Engelhardt JF, Zandi E (2005). Iron-mediated H2O2 production as a mechanism for cell type-specific inhibition of tumor necrosis factor alpha-induced but not interleukin-1beta-induced IkappaB kinase complex/nuclear factor-kappaB activation.. J Biol Chem.

[25] Shen SC, Yang LY, Lin HY, Wu CY, Su TH (2008). Reactive oxygen species-dependent HSP90 protein cleavage participates in arsenical As(+3)- and MMA(+3)-induced apoptosis through inhibition of telomerase activity via JNK activation.. Toxicol Appl Pharmacol.

[26] Halliwell B, Gutteridge JM (1992). Biologically relevant metal ion-dependent hydroxyl radical generation. An update.. FEBS Lett.

[27] Soti C, Racz A, Csermely P (2002). A Nucleotide-dependent molecular switch controls ATP binding at the C-terminal domain of Hsp90. N-terminal nucleotide binding unmasks a C-terminal binding pocket.. J Biol Chem.

[28] Soti C, Vermes A, Haystead TA, Csermely P (2003). Comparative analysis of the ATP-binding sites of Hsp90 by nucleotide affinity cleavage: a distinct nucleotide specificity of the C-terminal ATP-binding site.. Eur J Biochem.

[29] Burton K (1959). Formation constants for the complexes of adenosine di- or tri-phosphate with magnesium or calcium ions.. Biochem J.

[30] Goucher CR, Taylor JF (1964). Compounds of Ferric Iron with Adenosine Triphosphate and Other Nucleoside Phosphates.. J Biol Chem.

[31] Samuni A, Aronovitch J, Godinger D, Chevion M, Czapski G (1983). On the cytotoxicity of vitamin C and metal ions. A site-specific Fenton mechanism.. Eur J Biochem.

[32] Bergerat A, de Massy B, Gadelle D, Varoutas PC, Nicolas A (1997). An atypical topoisomerase II from Archaea with implications for meiotic recombination.. Nature.

[33] Gutteridge JM (1987). Ferrous-salt-promoted damage to deoxyribose and benzoate. The increased effectiveness of hydroxyl-radical scavengers in the presence of EDTA.. Biochem J.

[34] Trachootham D, Alexandre J, Huang P (2009). Targeting cancer cells by ROS-mediated mechanisms: a radical therapeutic approach?. Nat Rev Drug Discov.

[35] Wondrak GT (2009). Redox-directed cancer therapeutics: molecular mechanisms and opportunities.. Antioxid Redox Signal.

[36] Ye X, Fels D, Tovmasyan A, Aird KM, Dedeugd C (2011). Cytotoxic effects of Mn(III) N-alkylpyridylporphyrins in the presence of cellular reductant, ascorbate.. Free Radic Res.

