# Supplementary figures and images for: Hsp90 Is Cleaved by Reactive Oxygen Species at a Highly Conserved N-Terminal Amino Acid Motif
Source: PLoS One. 2012 Jul 27;7(7):e40795. doi: 10.1371/journal.pone.0040795 (PMC3407180; doi:10.1371/journal.pone.0040795)

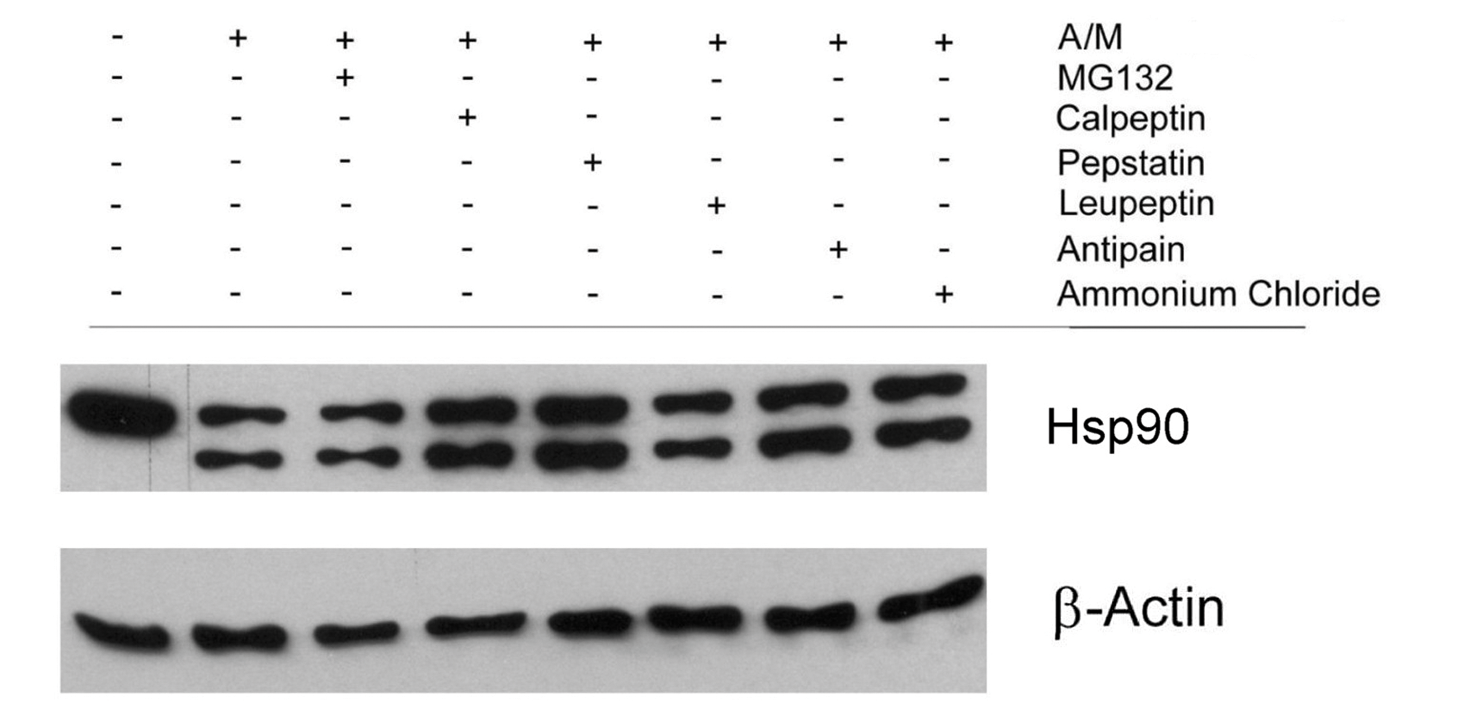

Supplement: Figure S1 — Hsp90 cleavage is not suppressed by protease inhibitors. K562 cells were preincubated for 1 h with the following compounds: MG132 (40 µM), calpeptin (50 µM), pepstatin (100 µM), antipain (50 µM), leupeptin (200 µM), and NH4Cl (5 mM), and then further exposed to A/M (2 mM/10 µM) for 2 h. Hsp90 was detected with an anti-C terminus antibody from Santa Cruz Biotechnology (Hsp90 α/β, clone F-8). (TIF) [file pone.0040795.s001.tif]

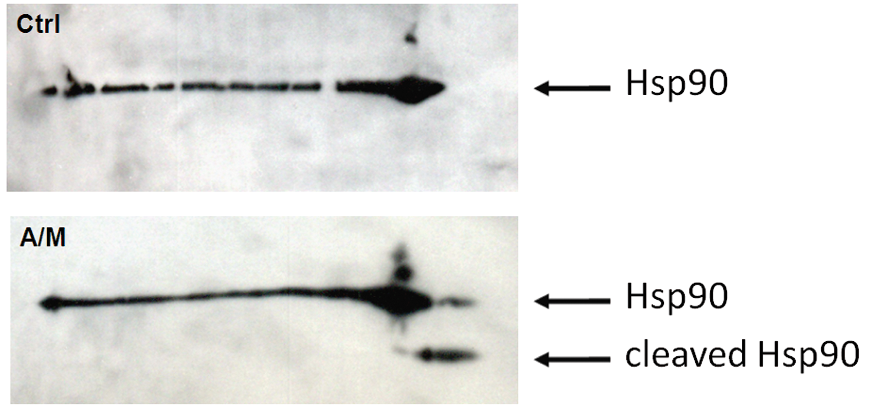

Supplement: Figure S2 — Detection of cleaved and uncleaved Hsp90 after 2D gel electrophoresis. Cells were incubated for 2 h in the absence (Ctrl) or in the presence of A/M (2 mM/10 µM). Cells were lysed and samples were run on 2D gels, as described in the Material and Methods section. Proteins were then transferred to PVDF membranes and Hsp90 was detected with an anti-C terminus antibody from Santa Cruz Biotechnology (Hsp90 α/β, clone F-8). (TIF) [file pone.0040795.s002.tif]
