# Supplementary material for: Hsp90 Is Cleaved by Reactive Oxygen Species at a Highly Conserved N-Terminal Amino Acid Motif
Source: PLoS One. 2012 Jul 27;7(7):e40795. doi: 10.1371/journal.pone.0040795 (PMC3407180; doi:10.1371/journal.pone.0040795)
Supplement: Table S1 — High-resolution UHR-Qq-TOF mass measurements of peptides formed by the proteolytic digestion of non-cleaved Hsp90. (DOC) [file pone.0040795.s003.doc]

**Table S1**

High-resolution UHR-Qq-TOF mass measurements of peptides formed by the proteolytic digestion of non-cleaved Hsp90.

| **Measured mass (m/z)** | **Theoretical mass** | **z** | **Error [ppm]** | **Scores** | **Sequence** | **Modifications** | **Range** |
| --- | --- | --- | --- | --- | --- | --- | --- |
| 834.37080 | 1666.71109 | 2 | 9.56 | 77.5 (M:77.5) | M.PEEVHHGEEEVETF.A |  | 2 – 15 |
| 677.40290 | 676.39081 | 1 | 7.10 | 25.8 (M:25.8) | K.EIFLR.E |  | 37 – 41 |
| 638.33050 | 1274.63541 | 2 | 8.64 | 60.7 (M:60.7) | R.ELISNASDALDK.I |  | 42 – 53 |
| 772.92340 | 1543.82059 | 2 | 7.54 | 101.7 (M:101.7) | R.ELISNASDALDKIR.Y |  | 42 – 55 |
| 733.39190 | 1464.75726 | 2 | 8.17 | 102.1 (M:102.1) | L.ISNASDALDKIRY.E |  | 44 – 56 |
| 1039.50310 | 1038.48696 | 1 | 8.53 | 61.6 (M:61.6) | R.YESLTDPSK.L |  | 56 – 64 |
| 770.38670 | 1538.74642 | 2 | 8.07 | 101.3 (M:101.3) | R.YESLTDPSKLDSGK.E |  | 56 – 69 |
| 495.26560 | 988.50769 | 2 | 9.04 | 43.9 (M:43.9) | Y.ESLTDPSKL.D |  | 57 – 65 |
| 782.94390 | 1563.86206 | 2 | 7.15 | 48.1 (M:48.1) | K.ELKIDIIPNPQER.T |  | 70 – 82 |
| 768.94660 | 1535.86714 | 2 | 7.48 | 73.6 (M:73.6) | L.KIDIIPNPQERTL.T |  | 72 – 84 |
| 597.83220 | 1193.64044 | 2 | 7.87 | 59.7 (M:59.7) | K.IDIIPNPQER.T |  | 73 – 82 |
| 683.37380 | 1364.72212 | 2 | 8.00 | 83.0 (M:83.0) | R.TLTLVDTGIGMTK.A | Oxidation: 11 | 83 – 95 |
| 675.37560 | 1348.72720 | 2 | 6.99 | 81.3 (M:81.3) | R.TLTLVDTGIGMTK.A |  | 83 – 95 |
| 621.86100 | 1241.69795 | 2 | 7.64 | 67.2 (M:67.2) | K.ADLINNLGTIAK.S |  | 96 – 107 |
| 857.40160 | 1712.77496 | 2 | 7.98 | 73.7 (M:73.7) | F.MEALQAGADISMIGQF.G | Oxidation: 1, 12 | 114 – 129 |
| 700.71040 | 2099.08988 | 3 | 9.27 | 31.6 (M:31.6) | Y.LVAEKVVVITKHNDDEQY.A |  | 138 – 155 |
| 663.01510 | 1986.00582 | 3 | 8.87 | 23.2 (M:23.2) | L.VAEKVVVITKHNDDEQY.A |  | 139 – 155 |
| 748.72120 | 2243.12225 | 3 | 8.69 | 28.5 (M:28.5) | L.VAEKVVVITKHNDDEQYAW.E |  | 139 – 157 |
| 658.45480 | 657.44251 | 1 | 7.61 | 32.1 (M:32.1) | K.VVVITK.H |  | 143 – 148 |
| 752.66390 | 2254.95155 | 3 | 8.11 | 75.4 (M:75.4) | K.HNDDEQYAWESSAGGSFTVR.A |  | 149 – 168 |
| 640.37150 | 1918.07484 | 3 | 9.28 | 36.6 (M:36.6) | F.TVRADHGEPIGRGTKVIL.H |  | 166 – 183 |
| 476.23950 | 950.45699 | 2 | 7.83 | 37.5 (M:37.5) | R.ADHGEPIGR.G |  | 169 – 177 |
| 672.35800 | 2014.03712 | 3 | 7.46 | 104.3 (M:104.3) | K.VILHLKEDQTEYLEER.R |  | 181 – 196 |
| 581.77740 | 1161.53022 | 2 | 8.62 | 43.5 (M:43.5) | L.HLKEDQTEY.L |  | 184 – 192 |
| 656.29430 | 1310.56264 | 2 | 8.69 | 77.7 (M:77.7) | K.EDQTEYLEER.R |  | 187 – 196 |
| 576.28740 | 1150.55062 | 2 | 8.35 | 45.1 (M:45.1) | K.YIDQEELNK.T |  | 276 – 284 |
| 783.39520 | 2347.14444 | 3 | 8.23 | 34.7 (M:34.7) | L.NKTKPIWTRNPDDITQEEY.G |  | 283 – 301 |
| 451.27000 | 900.51814 | 2 | 8.10 | 35.0 (M:35.0) | K.TKPIWTR.N |  | 285 – 291 |
| 907.40520 | 1812.78024 | 2 | 8.60 | 95.4 (M:95.4) | W.TRNPDDITQEEYGEF.Y |  | 290 – 304 |
| 924.40970 | 1846.78974 | 2 | 8.17 | 117.0 (M:117.0) | R.NPDDITQEEYGEFYK.S |  | 292 – 306 |
| 764.38210 | 1526.73652 | 2 | 8.59 | 85.9 (M:85.9) | K.SLTNDWEDHLAVK.H |  | 307 – 319 |
| 908.44360 | 907.42871 | 1 | 8.38 | 21.7 (M:21.7) | F.SVEGQLEF.R |  | 322 – 329 |
| 674.84120 | 1347.65715 | 2 | 7.93 | 31.8 (M:31.8) | K.HFSVEGQLEFR.A |  | 320 – 330 |
| 415.27140 | 828.52216 | 2 | 7.33 | 60.0 (M:60.0) | R.ALLFIPR.R |  | 331 – 337 |
| 618.82690 | 1235.62987 | 2 | 7.58 | 43.4 (M:43.4) | R.RAPFDLFENK.K |  | 338 – 347 |
| 540.77590 | 1079.52876 | 2 | 7.85 | 39.1 (M:39.1) | R.APFDLFENK.K |  | 339 – 347 |
| 787.42180 | 1572.81477 | 2 | 9.06 | 59.5 (M:59.5) | L.NFIRGVVDSEDLPL.N |  | 375 – 388 |
| 656.86490 | 1311.70343 | 2 | 8.99 | 65.5 (M:65.5) | F.IRGVVDSEDLPL.N |  | 377 – 388 |
| 757.40260 | 1512.77839 | 2 | 8.09 | 99.9 (M:99.9) | R.GVVDSEDLPLNISR.E |  | 379 – 392 |
| 788.43400 | 1574.84503 | 2 | 5.34 | 61.1 (M:61.1) | L.NISREMLQQSKIL.K | Oxidation: 6 | 389 – 401 |
| 599.27270 | 1196.51971 | 2 | 9.29 | 33.0 (M:33.0) | F.SELAEDKENY.K |  | 417 – 426 |
| 891.43170 | 890.41742 | 1 | 7.86 | 45.1 (M:45.1) | K.FYEAFSK.N |  | 429 – 435 |
| 565.65620 | 1693.93360 | 3 | 7.76 | 21.1 (M:21.1) | L.KLGIHEDSTNRRRL.S |  | 438 – 451 |
| 571.28850 | 1140.55235 | 2 | 8.84 | 68.6 (M:68.6) | K.LGIHEDSTNR.R |  | 439 – 448 |
| 443.78030 | 885.53960 | 2 | 7.26 | 30.5 (M:30.5) | R.RLSELLR.Y |  | 450 – 456 |
| 365.72940 | 729.43849 | 2 | 7.87 | 44.2 (M:44.2) | R.LSELLR.Y |  | 451 – 456 |
| 726.32580 | 2175.93788 | 3 | 8.12 | 102.6 (M:102.6) | R.YHTSQSGDEMTSLSEYVSR.M |  | 457 – 475 |
| 731.65810 | 2191.93279 | 3 | 8.97 | 100.3 (M:100.3) | R.YHTSQSGDEMTSLSEYVSR.M | Oxidation: 10 | 457 – 475 |
| 836.35050 | 1670.67299 | 2 | 8.04 | 95.5 (M:95.5) | Y.HTSQSGDEMTSLSEY.V |  | 458 – 472 |
| 654.77750 | 1307.52996 | 2 | 8.01 | 36.4 (M:36.4) | Y.HTSQSGDEMTSL.S | Oxidation: 9 | 458 – 469 |
| 824.92910 | 1647.82904 | 2 | 8.85 | 23.0 (M:23.0) | Y.VSRMKETQKSIYY.I | Oxidation: 4 | 473 – 485 |
| 495.93330 | 1484.76571 | 3 | 8.31 | 25.9 (M:25.9) | Y.VSRMKETQKSIY.Y | Oxidation: 4 | 473 – 484 |
| 580.80030 | 1159.57610 | 2 | 8.56 | 48.8 (M:48.8) | K.SIYYITGESK.E |  | 482 – 491 |
| 822.40630 | 1642.78387 | 2 | 8.62 | 106.6 (M:106.6) | Y.YITGESKEQVANSAF.V |  | 485 – 499 |
| 740.87440 | 1479.72054 | 2 | 9.25 | 64.1 (M:64.1) | Y.ITGESKEQVANSAF.V |  | 486 – 499 |
| 625.31770 | 1248.60986 | 2 | 8.78 | 99.1 (M:99.1) | K.EQVANSAFVER.V |  | 492 – 502 |
| 822.05830 | 2463.13380 | 3 | 7.81 | 34.1 (M:34.1) | R.GFEVVYMTEPIDEYCVQQLK.E | Carbamidomethyl: 15; Oxidation: 7 | 507 – 526 |
| 733.45140 | 732.43815 | 1 | 8.14 | 56.3 (M:56.3) | K.SLVSVTK.E |  | 532 – 538 |
| 772.87570 | 1543.72535 | 2 | 7.44 | 48.2 (M:48.2) | K.EGLELPEDEEEKK.K |  | 539 – 551 |
| 708.82830 | 1415.63038 | 2 | 8.23 | 67.7 (M:67.7) | K.EGLELPEDEEEK.K |  | 539 – 550 |
| 505.26440 | 1008.50625 | 2 | 7.91 | 35.5 (M:35.5) | K.AKFENLCK.L | Carbamidomethyl: 7 | 558 – 565 |
| 810.38740 | 809.37417 | 1 | 7.34 | 24.9 (M:24.9) | K.FENLCK.L | Carbamidomethyl: 5 | 560 – 565 |
| 745.45120 | 744.43815 | 1 | 7.74 | 20.0 (M:20.0) | K.EILDKK.V |  | 569 – 574 |
| 510.64290 | 1528.89369 | 3 | 8.60 | 37.1 (M:37.1) | L.DKKVEKVTISNRL.V |  | 572 – 584 |
| 345.20320 | 688.38679 | 2 | 7.33 | 26.9 (M:26.9) | K.VTISNR.L |  | 578 – 583 |
| 632.75850 | 1263.49338 | 2 | 7.16 | 30.9 (M:30.9) | R.DNSTMGYMMAK.K | Oxidation: 8 | 613 – 623 |
| 624.76060 | 1247.49846 | 2 | 6.55 | 48.7 (M:48.7) | R.DNSTMGYMMAK.K |  | 613 – 623 |
| 478.52030 | 1910.03740 | 4 | 7.68 | 51.0 (M:51.0) | K.KHLEINPDHPIVETLR.Q |  | 624 – 639 |
| 891.98520 | 1781.94243 | 2 | 7.52 | 94.4 (M:94.4) | K.HLEINPDHPIVETLR.Q |  | 625 – 639 |
| 714.08060 | 2139.20117 | 3 | 8.78 | 74.8 (M:74.8) | L.RQKAEADKNDKAVKDLVVL.L |  | 639 - 657 |
