# Supplementary material for: Hsp90 Is Cleaved by Reactive Oxygen Species at a Highly Conserved N-Terminal Amino Acid Motif
Source: PLoS One. 2012 Jul 27;7(7):e40795. doi: 10.1371/journal.pone.0040795 (PMC3407180; doi:10.1371/journal.pone.0040795)
Supplement: Table S2 — High-resolution UHR-Qq-TOF mass measurements of peptides formed by the proteolytic digestion of the 70 kDa C-terminal fragment of cleaved Hsp90. (DOC) [file pone.0040795.s004.doc]

**Table S2**

High-resolution UHR-Qq-TOF mass measurements of peptides formed by the proteolytic digestion of the 70 kDa C-terminal fragment of cleaved Hsp90.

| **Measured mass (m/z)** | **Theoretical mass** | **z** | **Error [ppm]** | **Scores** | **Sequence** | **Modifications** | **Range** |
| --- | --- | --- | --- | --- | --- | --- | --- |
| 700.70970 | 2099.08988 | 3 | 8.27 | 29.0 (M:29.0) | Y.LVAEKVVVITKHNDDEQY.A |  | 138 - 155 |
| 640.37090 | 1918.07484 | 3 | 8.34 | 23.6 (M:23.6) | F.TVRADHGEPIGRGTKVIL.H |  | 166 - 183 |
| 672.35370 | 2014.03712 | 3 | 1.07 | 91.5 (M:91.5) | K.VILHLKEDQTEYLEER.R |  | 181 - 196 |
| 581.77670 | 1161.53022 | 2 | 7.42 | 33.6 (M:33.6) | L.HLKEDQTEY.L |  | 184 - 192 |
| 656.28920 | 1310.56264 | 2 | 0.92 | 79.2 (M:79.2) | K.EDQTEYLEER.R |  | 187 - 196 |
| 576.28280 | 1150.55062 | 2 | 0.37 | 42.3 (M:42.3) | K.YIDQEELNK.T |  | 276 - 284 |
| 907.40510 | 1812.78024 | 2 | 8.49 | 67.9 (M:67.9) | W.TRNPDDITQEEYGEF.Y |  | 290 - 304 |
| 924.40400 | 1846.78974 | 2 | 2.01 | 118.3 (M:118.3) | R.NPDDITQEEYGEFYK.S |  | 292 - 306 |
| 764.37590 | 1526.73652 | 2 | 0.47 | 59.6 (M:59.6) | K.SLTNDWEDHLAVK.H |  | 307 - 319 |
| 415.26790 | 828.52216 | 2 | -1.10 | 41.1 (M:41.1) | R.ALLFIPR.R |  | 331 - 337 |
| 618.82270 | 1235.62987 | 2 | 0.79 | 31.1 (M:31.1) | R.RAPFDLFENK.K |  | 338 - 347 |
| 787.42120 | 1572.81477 | 2 | 8.30 | 62.4 (M:62.4) | L.NFIRGVVDSEDLPL.N |  | 375 - 388 |
| 656.86480 | 1311.70343 | 2 | 8.84 | 56.8 (M:56.8) | F.IRGVVDSEDLPL.N |  | 377 - 388 |
| 757.39780 | 1512.77839 | 2 | 1.76 | 107.6 (M:107.6) | R.GVVDSEDLPLNISR.E |  | 379 - 392 |
| 788.43490 | 1574.84503 | 2 | 6.48 | 29.4 (M:29.4) | L.NISREMLQQSKIL.K | Oxidation: 6 | 389 - 401 |
| 599.27180 | 1196.51971 | 2 | 7.79 | 28.4 (M:28.4) | F.SELAEDKENY.K |  | 417 - 426 |
| 726.32040 | 2175.93788 | 3 | 0.69 | 61.6 (M:61.6) | R.YHTSQSGDEMTSLSEYVSR.M |  | 457 - 475 |
| 731.65240 | 2191.93279 | 3 | 1.17 | 122.8 (M:122.8) | R.YHTSQSGDEMTSLSEYVSR.M | Oxidation: 10 | 457 - 475 |
| 844.34750 | 1686.66791 | 2 | 7.42 | 86.0 (M:86.0) | Y.HTSQSGDEMTSLSEY.V | Oxidation: 9 | 458 - 472 |
| 743.39580 | 1484.76571 | 2 | 7.62 | 23.8 (M:23.8) | Y.VSRMKETQKSIY.Y | Oxidation: 4 | 473 - 484 |
| 580.79540 | 1159.57610 | 2 | 0.12 | 67.5 (M:67.5) | K.SIYYITGESK.E |  | 482 - 491 |
| 822.40580 | 1642.78387 | 2 | 8.01 | 67.1 (M:67.1) | Y.YITGESKEQVANSAF.V |  | 485 - 499 |
| 740.87310 | 1479.72054 | 2 | 7.50 | 48.3 (M:48.3) | Y.ITGESKEQVANSAF.V |  | 486 - 499 |
| 625.31290 | 1248.60986 | 2 | 1.11 | 80.5 (M:80.5) | K.EQVANSAFVER.V |  | 492 - 502 |
| 1232.57650 | 2463.13380 | 2 | 1.89 | 38.1 (M:38.1) | R.GFEVVYMTEPIDEYCVQQLK.E | Carbamidomethyl: 15; Oxidation: 7 | 507 - 526 |
| 708.82330 | 1415.63038 | 2 | 1.17 | 27.7 (M:27.7) | K.EGLELPEDEEEK.K |  | 539 - 550 |
| 510.64270 | 1528.89369 | 3 | 8.21 | 27.4 (M:27.4) | L.DKKVEKVTISNRL.V |  | 572 - 584 |
| 478.51660 | 1910.03740 | 4 | -0.05 | 74.2 (M:74.2) | K.KHLEINPDHPIVETLR.Q |  | 624 - 639 |
| 714.07990 | 2139.20117 | 3 | 7.80 | 44.8 (M:44.8) | L.RQKAEADKNDKAVKDLVVL.L |  | 639 - 657 |
